# Commentary: Team Cognition in Sport: How Current Insights Into How Teamwork Is Achieved in Naturalistic Settings Can Lead to Simulation Studies

**DOI:** 10.3389/fpsyg.2020.00081

**Published:** 2020-02-07

**Authors:** Pamela Richards, Dave Collins

**Affiliations:** ^1^Institute of Coaching and Performance, University of Central Lancashire, Preston, United Kingdom; ^2^Moray House School of Education and Sport, ISPEHS, University of Edinburgh, Edinburgh, United Kingdom

**Keywords:** decision making, naturalistic decision making (NDM), team sports, team cognition, teamwork

## Introduction

The paper by Bourbousson et al. (2019) prompts discussion surrounding the complexity of team cognitions in sport. The authors rightly recognize the importance of naturalistic settings in developing cognitive components relating to teamwork, and the title suggests that enhancing understanding of the setting can facilitate simulation studies. We applaud their efforts to drive this important area, especially since they go beyond the perception-alone focus which has recently come to dominate the area. Importantly, however, although we do not refute the connection between naturalistic settings and growing interest in simulation studies, we have concerns regarding the theoretical approaches they used to inform understanding of team cognitions, and the subsequent methods suggested. Accordingly, we outline two key concerns and provide evidence that, although to some extent we support the authors' argument that investigating and developing team cognitions (teamwork) is complex and requires the integration of multiple theories, the theoretical approaches, and subsequent methods suggested may be limited.

Our first concern relates to the authors' delimited application of NDM as a theoretical paradigm to examine team cognition. To truly understand team cognitive processes, it is essential that teamwork is examined within the naturalistic setting. Exploration of team cognitive processes outside of the performance setting (in isolation of the playing context) risks the over inflation of cognitive aspects (Williams, 2009). The point is that talking about it is not necessarily the same as doing it! Indeed, the use of Shared Mental Models (SMMs—Richards et al., 2016) often acts a priori to direct actions along certain lines, even if players don't bother to articulate their rejection of these options. Furthermore, basing arguments solely or overly on data around on-field verbal interactions, whilst not pursuing the mental models developed off-field which also drive on-field decision making, would seem an inappropriate delimitation. For example, our own work shows how off-field activity, slower overt debate coupled with more time pressured activities, can be used to develop and then drive on-field behavior. Importantly, this on-field behavior will often occur with less, or even no overt verbalization between players. As a further consequence, and against the arguments of Bourbosson et al. that only a limited number of players need to be involved in this, over verbalizations may bear little or no relation to actually who, or what, is driving the behavior. In short, research needs to look at *what* is happening on the pitch but also *why* it is happening, with the second element almost necessitating off-field consideration.

In an attempt to address this “combination,” NDM researchers seek to investigate how experts perform tasks in dynamic environments that have ill-structured problems, changing objectives, time constraints, include multiple players and are influenced by organizational goals (Klein, 2008): all characteristics representative of team sport (Richards et al., 2009). Regrettably, such real-world approaches involve a high volume of contextual information which have, until recently, been neglected (Richards et al., 2016). Although recognition of the significance of the naturalistic setting is shared with Bourbosson et al., we feel their interpretation is limited with the exclusion of some key data sources.

Theories emerging from the NDM paradigm such as Recognition Primed Decision Making (RPD; Klein, 1998), Situational Awareness (SA; Endsley and Garland, 2008), and Sensemaking (Klein et al., 2007) make a valuable contribution individually to enhance our understanding of team cognitive process in a naturalistic setting. However, when integrated *collectively*, they provide a comprehensive justification for the possible approaches through which team cognitive processes can be developed and explored. Furthermore, Klein (2000) has identified five key cognitive processes involved in teamwork. These include control of attention, shared situational awareness, shared mental-models, application of strategies/heuristics, and metacognitions. This commentary does not refute the contribution from other theoretical approaches such as neuroscience (Aglioti et al., 2008), motor control (Starkes and Ericson, 2003; Williams, 2009) and other theories referred to by Bourbosson et al. All have enhanced our understanding of cognitive processes. However, we would argue such work is limited as it has ignored the integration of *both* psychomotor *and* psychosocial components which are required in sports teams' cognitive processes (Richards et al., 2009, 2012, 2016). Such work examines performers' cognitive processes in a range of sports (hockey, Richards et al., 2009; netball, Richards et al., 2012), team coaches' and coach developers (Abraham et al., 2009), and sports officials (Mascarenhas et al., 2005). Thus, as a collective, these studies illustrate the important interplay between psychomotor and psychosocial elements of team cognitions in a naturalistic setting.

Our second concern rests with the limited methodological approaches suggested for working within a naturalistic setting. As stated above, and demonstrated in the body of work presented, in-action decision-making is significantly (and, we suggest, best) driven by mental models developed away from the field. Consequently, it is impossible to consider team cognitions in sport without drawing on Cognitive Task Analysis (CTA), a methodology which has emerged from the NDM research community. Relying on one methodological approach alone risks presenting a biased and distorted understanding of teamwork. By understanding what combination of cognitive and social processes are involved in naturalistic teamwork, appropriate methodologies (from the same paradigmatic approach) can be employed to examine the specific processes, thus advancing our understanding of how sports teams perform. CTA involves a range of techniques concerned with the elicitation of knowledge of experts (Hoffman and Militello, 2009) in a naturalistic setting. Such techniques have proved successful in capturing the unobservable cognitive processes, decisions, and judgements embedded in expert performances (Chipman et al., 2000) and subsequently, in the training/development of cognitive team skills (Richards et al., 2012). Using multiple CTA methods (for example, Critical Decision Method, Applied Cognitive Task Analysis, and Concept Mapping) in one “combinatorics” approach, allows the exploration of different cognitive and social processes (Hoffman and Militello, 2009) to be examined within team sports (Richards et al., 2016).

To conclude, it is paramount that when examining cognitive processes within a naturalistic setting, a *full* range of theoretical approaches from NDM are utilized. The methodologies employed by NDM researchers are designed specifically to examine the dynamic naturalistic setting—matching epistemological and methodological assumptions. Application of CTA allows researchers to focus on understanding how experts perform a cognitive task in a naturalistic setting (Gore and McAndrew, 2009) driven by *in-situ* and a priori inputs. Without the inclusion of this breadth of data, only a partial picture can possibly emerge.

## Author Contributions

PR wrote the article with additional comments and revisions by DC.

### Conflict of Interest

The authors declare that the research was conducted in the absence of any commercial or financial relationships that could be construed as a potential conflict of interest.
